# Detection and function of an intramolecular disulfide bond in the pH-responsive CadC of *Escherichia coli*

**DOI:** 10.1186/1471-2180-11-74

**Published:** 2011-04-12

**Authors:** Larissa Tetsch, Christiane Koller, Alexandra Dönhöfer, Kirsten Jung

**Affiliations:** 1 Munich Center for integrated Protein Science (CiPSM) at the Department of Biology I, Microbiology, Ludwig-Maximilians-Universität München, Grosshaderner Straße 2-4, 82152 Martinsried, Germany; 2 Lehrstuhl für Physiologische Chemie, Veterinärwissenschaftliches Department, Tierärztliche Fakultät der Ludwig-Maximilians-Universität München, Veterinärstr. 13, 80539 Munich, Germany; 3 Gene Center Munich, Department of Biochemistry, Ludwig-Maximilians-Universität München, Feodor-Lynen-Str. 25, 81377 Munich, Germany

## Abstract

**Background:**

In an acidic and lysine-rich environment *Escherichia coli *induces expression of the *cadBA *operon which encodes CadA, the lysine decarboxylase, and CadB, the lysine/cadaverine antiporter. *cadBA *expression is dependent on CadC, a membrane-integrated transcriptional activator which belongs to the ToxR-like protein family. Activation of CadC requires two stimuli, lysine and low pH. Whereas lysine is detected by an interplay between CadC and the lysine-specific transporter LysP, pH alterations are sensed by CadC directly. Crystal structural analyses revealed a close proximity between two periplasmic cysteines, Cys208 and Cys272.

**Results:**

Substitution of Cys208 and/or Cys272 by alanine resulted in CadC derivatives that were active in response to only one stimulus, either lysine or pH 5.8. Differential *in vivo *thiol trapping revealed a disulfide bond between these two residues at pH 7.6, but not at pH 5.8. When Cys208 and Cys272 were replaced by aspartate and lysine, respectively, virtually wild-type behavior was restored indicating that the disulfide bond could be mimicked by a salt bridge.

**Conclusion:**

A disulfide bond was found in the periplasmic domain of CadC that supports an inactive state of CadC at pH 7.6. At pH 5.8 disulfide bond formation is prevented which transforms CadC into a semi-active state. These results provide new insights into the function of a pH sensor.

## Background

*Escherichia coli *uses several strategies to maintain a neutral cytoplasmic pH in an acidic environment helping the bacterium to survive under this unfavorable condition. A number of genes is induced upon exposure of cells to low pH, among them the genes for the degradative amino acid decarboxylase systems *adi*, *gad *and *cad*. The Cad system consists of the cytoplasmic protein CadA and the transmembrane proteins CadB and CadC [1]. CadA is a lysine decarboxylase that catalyzes decarboxylation of lysine to cadaverine whereby one proton is consumed resulting in a relief of the intracellular acid stress. The alkaline product cadaverine is concomitantly excreted by the lysine/cadaverine antiporter CadB [2,3]. The genes *cadA *and *cadB *are organized in an operon [3,4], which is under the control of the P_*Cad *_promoter. Expression of the *cadBA *operon is induced after external acidification, and simultaneous presence of extracellular lysine. CadC is the positive regulator of *cadBA *expression [5], and binds to two sites within the *cadBA *promoter [6].

*cadC *is located upstream of the *cadBA *operon and encodes a 58 kDa inner membrane protein. CadC, a member of the ToxR-like transcriptional activators [7], consists of a cytoplasmic N-terminal domain (amino acids 1-158), a single transmembrane domain (amino acids 159-187), and a periplasmic C-terminal domain (amino acids 188-512) [5,8]. The cytoplasmic domain shows sequence similarity to the RO_II_-subgroup of DNA-binding domains of response regulators [5]. However, contrary to prototypical response regulators [9] signal transduction in CadC functions without phosphorylation. Thus, CadC and all other ToxR-like proteins represent a one-component stimulus-response system.

Based on CadC derivatives with altered sensing properties due to single amino acid replacements within the periplasmic domain, it was suggested that this domain is the signal input domain [8]. Recently, it became clear that CadC senses alterations of the external pH directly [10], but lysine is sensed only indirectly. The lysine-dependent regulation of CadC is exerted by an interplay with the lysine permease LysP, and it is proposed that in the absence of lysine, CadC is inactivated by an interaction with LysP [11].

Here, we investigated the role of the three cysteine residues in CadC. The best investigated member of the ToxR-like protein family, ToxR of *Vibrio cholerae*, contains two cysteines within the periplasmic domain. These cysteines were found to be involved in the formation of an intramolecular disulfide bond but also in the formation of intermolecular disulfide bonds between two ToxR molecules and between ToxR and a second transmembrane protein, ToxS [12,13]. Although it was shown that ToxR binds to the DNA only in a dimeric form [7], ToxR oligomerization *in vivo *was independent of environmental changes [14], and thus evidence for the functional importance of the cysteines in ToxR is still lacking. Our studies indicated that a disulfide bond within the periplasmic domain of CadC is formed at pH 7.6, but these cysteines are in the reduced state at pH 5.8. These results give new insights into the switch between inactive and active states of a pH-responsive protein.

## Results

### Two periplasmic cysteines are important for the regulation of CadC activity

CadC of *E. coli *contains three cysteine residues, one in the transmembrane domain (C172), and two in the periplasmic domain (C208 and C272). Amino acid alignment of CadC from all available sequences indicated that C172 is found only in a few species, whereas the two periplasmic cysteines are well conserved in the order of Enterobacteriales (data not shown). In addition, the crystal structure of the periplasmic domain of CadC depicted a close proximity between C208 and C272 [15] predicting an intramolecular disulfide bond. Thus, the role of the cysteines in CadC was studied in detail.

**Figure 1:**
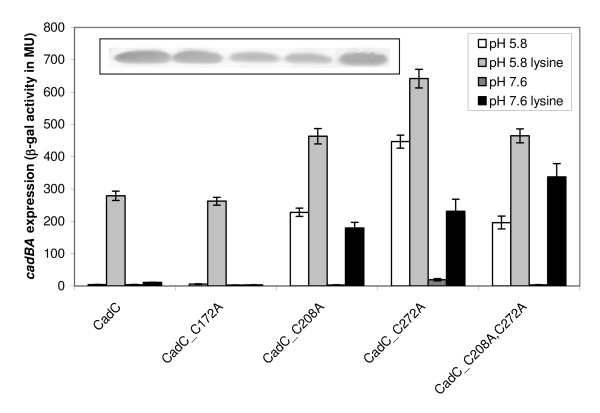
**Influence of cysteine replacements in CadC on *cadBA *expression**. Reporter gene assays were performed with *E. coli *EP314 (*cadC*::Tn*10*; *cadA'*::*lacZ *fusion) which was complemented with plasmid-encoded *cadC *or the indicated *cadC *derivatives. Cells were cultivated under microaerobic conditions in minimal medium at pH 5.8 or pH 7.6 in the presence or absence of 10 mM lysine at 37°C to mid-logarithmic growth phase, and harvested by centrifugation. The activity of the reporter enzyme β-galactosidase was determined [43] and served as a measurement for *cadBA *expression. Error bars indicate standard deviations of the mean for at least three independent experiments. To analyze production and membrane integration of the CadC derivatives, Western blot analysis of membrane fractions from *E. coli *BL21(DE3)pLysS transformed with plasmids encoding either wild-type or CadC derivatives was performed (inset). Each lane contains 25 μg of membrane protein (CadC derivatives are in the same order as in the graph). CadC was detected by a monoclonal mouse antibody against the His-Tag and an alkaline phosphatase coupled anti-mouse antibody.

First, each cysteine in CadC was replaced with alanine, and the resulting derivatives CadC_C172A, CadC_C208A, CadC_C272A and CadC_C208A,C272A were used for complementation of the *E. coli *EP314 reporter strain (*cadC*::Tn*10*, *cadA'*::*lacZ*). β-Galactosidase activities were determined as a measurement for *cadBA *expression. CadC_C172A with a replacement of the cysteine in the transmembrane domain retained the activity pattern of wild-type CadC with induction of *cadBA *expression only at pH 5.8 in the presence of lysine (Figure 1). In contrast, replacement of cysteines at positions 208 and 272 in the periplasmic domain either alone or in combination resulted in CadC derivatives for which one stimulus was sufficient to activate *cadBA *expression (Figure 1). Specifically, cells expressing these derivatives induced *cadBA *expression at pH 5.8 regardless of the presence of lysine, and also at pH 7.6 when lysine was present. In general, β-galactosidase activities were significantly higher for these derivatives compared to the wild-type. Besides, a comparison of the activities in response to one or two stimuli revealed that the induction level significantly increased when cells expressing these derivatives were exposed to both stimuli (Figure 1). All CadC derivatives analyzed in reporter gene assays were produced and found to be membrane-integrated as the wild-type protein (Figure 1). In consequence, C208 and C272 are important for the regulation of CadC activity.

In order to detect intermolecular disulfide bonds, membrane vesicles containing wild-type CadC or CadC derivatives with cysteine replacements were treated with copper phenanthroline, a Cys null crosslinker. Subsequent Western blot analysis revealed that in case of wild-type CadC and CadC with a single Cys at position 172, a fraction of the protein was transformed into an oligomeric form which might be related to the formation of an intermolecular disulfide bond at position 172 (data not shown). Since replacement of Cys172 was without effect on the CadC-mediated *cadBA *expression (Figure 1), it is concluded that an intermolecular disulfide bond is without functional importance for CadC.

### An intramolecular disulfide bond between C208 and C272 is found at pH 7.6 *in vivo*

To analyze whether a disulfide bond is formed in CadC, an *in vivo *differential thiol trapping approach with iodoacetamide and PEG-maleimide was used [16]. For simplification, these studies were performed with CadC_C172A which contains only the two periplasmic cysteines. The method is based on the fact that both iodoacetamide and PEG-maleimide react only with free thiol groups. First, *E. coli *cells producing CadC_C172A were labeled with iodoacetamide during growth at pH 7.6 or pH 5.8. Subsequently, free iodoacetamide was removed, and all disulfide bonds were reduced by treatment with dithiothreitol (DTT). Free thiol groups were labeled with PEG-maleimide in a second step. In consequence, only cysteines that are present in an oxidized form and thus protected from iodoacetamide labeling in the first step, are labeled with PEG-maleimide resulting in a detectable increase of the molecular weight.

**Figure 2:**
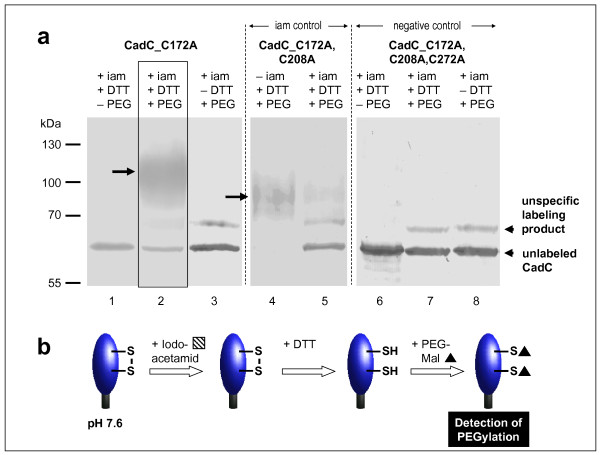
***In vivo *monitoring of the thiol/disulfide state of the periplasmic cysteines of CadC at pH 7.6 (a) and illustration of the results (b)**. (a) CadC_C172A, CadC_C172A,C208A or CadC_C172A,C208A,C272A (cysteine-free CadC) were overproduced in *E. coli *BL21(DE3)pLysS grown in phosphate buffered minimal medium at pH 7.6. To label free thiol groups irreversibly, 5 mM iodoacetamide was added directly to the living cells. After TCA precipitation and extensive washing, oxidized thiol groups were reduced by addition of 10 mM DTT in denaturing buffer. These reduced cysteines were then alkylated by addition of 10 mM PEG-maleimide. Samples were mixed with non-reducing SDS-sample buffer and 30 μg total cell protein were loaded onto 12.5% SDS-polyacrylamide gels. CadC was detected by Western blot analysis of the His-tagged proteins. Control experiments were done without DTT (lanes 3, 8) or PEG-mal (lanes 1, 6) or iam (lane 4). As a negative control the cysteine-free CadC derivative CadC_C172A,C208A,C272A was used. The iam control was performed with a CadC derivative that contains only one cysteine (CadC_C172A,C208A). iam = iodoacetamide, DTT = dithiothreitol, PEG = PEG-maleimide. (b) The results are schematically illustrated.

At pH 7.6 differential labeling of CadC_C172A clearly resulted in a labeling with PEG-maleimide (Figure 2). The band for unlabeled CadC decreased, and an additional higher molecular band appeared demonstrating labeling of C208 and C272 with PEG-maleimide (Figure 2a, lane 2). This additional band was only detectable when cells were treated with DTT (Figure 2a, lane 3 in comparison to lane 2). The PEG-ylated CadC_C172A runs as a smeared and broadened band which is probably due to the interaction between PEG and SDS [17]. Addition of PEG-maleimide (regardless of the treatment with DTT) resulted in an additional labeling product that also appeared in cells producing the cysteine-free CadC. Therefore, this signal can be regarded as unspecific labeling product which might be related to a reactivity of maleimide with other residues (e.g., lysine or tyrosine) in CadC (Figure 2a, lanes 2, 3, and 7, 8). Labeling of CadC_C172A with PEG-maleimide implies that iodoacetamide was unable to react with the periplasmic cysteines. To prove that this result was due to the presence of a disulfide bond that protected the cysteines from labeling, CadC_C172A,C208A was subjected to the same labeling procedure (Figure 2a, lanes 4 and 5). This CadC derivative contains one cysteine that should be labeled with iodoacetamide in the first labeling step. As expected this derivative was hardly PEG-ylated under this condition (Figure 2a, lane 5). In contrast, this protein was completely PEG-ylated when iodoacetamide was omitted in the first step (Figure 2a, lane 4). The PEG-ylated products (Figure 2a, lanes 2 and 4) differed in size because of the different number of cysteines that were accessible for labeling. These data clearly demonstrate the presence of a disulfide bond between C208 and C272 in the inactive state of CadC at pH 7.6 (Figure 2b).

**Figure 3:**
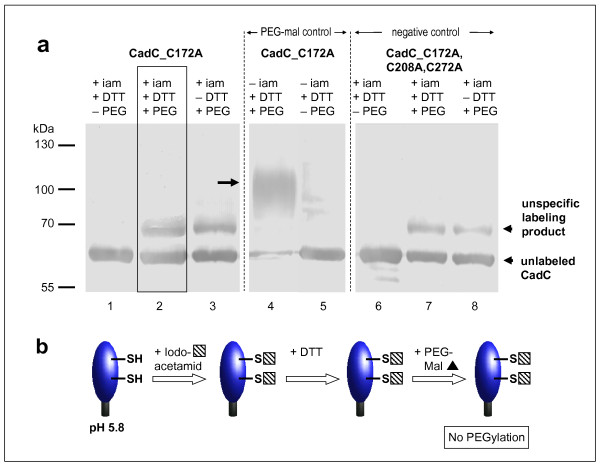
***In vivo *monitoring of the thiol/disulfide state of the periplasmic cysteines of CadC at pH 5.8 (a) and illustration of the results (b)**. (a) CadC_C172A or CadC_C172A,C208A,C272A were overproduced in *E. coli *BL21(DE3)pLysS grown in phosphate buffered minimal medium pH 5.8. The labeling procedure was essentially the same as described in Figure 2, with the difference that the alkylation time was prolonged. Control experiments were done without DTT (lanes 3, 8), or PEG-mal (lanes 1, 5, 6) or iam (lane 4, 5). As a negative control the cysteine-free CadC derivative CadC_C172A,C208A,C272A was used. iam = iodoacetamide, DTT = dithiothreitol, PEG = PEG-maleimide. (b) The results are schematically illustrated.

Since CadC becomes activated at low pH, the occurrence of the disulfide bond was also investigated under this condition (Figure 3). At pH 5.8 CadC_C172A was not labeled with PEG-maleimide (Figure 3a, lane 2). Addition of PEG-maleimide either in the absence or the presence of DTT produced only an unspecific band that was also observed for the cysteine-free CadC_C172A,C208A,C272A (Figure 3a, lanes 2, 3, and 7, 8). This result alludes to an efficient labeling of C208 and C272 with iodoacetamide in the first step, and implies that the periplasmic cysteines exist in a reduced form under acidic conditions. As a control, iodoacetamide was omitted and thereupon the typical PEG-maleimide labeling product appeared (Figure 3a, lane 4). Omittance of PEG-maleimide resulted in the disappearance of this band (Figure 3a, lane 5). These results reveal that PEG-ylation was possible under the used experimental conditions, but the cysteines in CadC_C172A were modified with iodoacetamide in the first step (Figure 3b). In conclusion, C208 and C272 are in a reduced form at low pH.

### The periplasmic disulfide bond can be mimicked by a salt bridge

**Figure 4:**
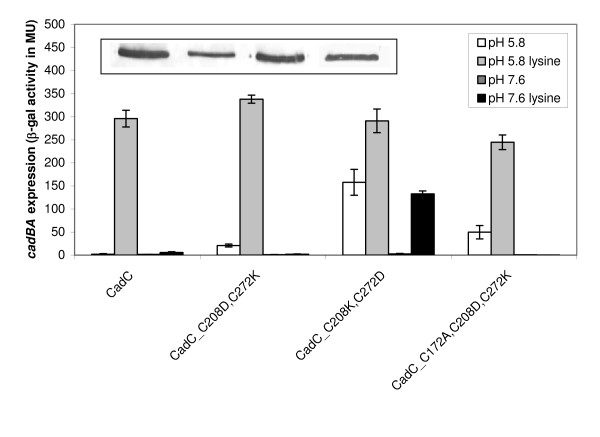
**Generation of a functional cysteine-free CadC by replacement of the disulfide bond forming cysteines with charged amino acids**. Reporter gene assays were performed with *E. coli *EP314 (*cadC*::Tn*10*; *cadA'*::*lacZ *fusion) which was complemented with plasmid-encoded *cadC *or the indicated *cadC *derivatives. Cells were cultivated under microaerobic conditions in minimal medium at pH 5.8 or pH 7.6 in the presence or absence of 10 mM lysine at 37°C to mid-logarithmic growth phase, and harvested by centrifugation. The activity of the reporter enzyme β-galactosidase was determined [43] and served as a measurement for *cadBA *expression. Error bars indicate standard deviations of the mean for at least three independent experiments. Western blot analysis (inset - see Figure 1 for details) of all CadC derivatives (same order as in the graph) indicates production and membrane insertion.

The results obtained with the labeling experiments indicate a disulfide bond under non-inducing conditions, but this bond is not formed at pH 5.8. In the next experiments we asked the question whether the disulfide bond could be mimicked by a salt bridge, which is strongly pH-dependent [18]. Therefore, C208 and C272 were replaced by lysine and aspartate in both combinations possible. Under non-inducing conditions (pH 7.6) these amino acids should be in their charged form, and thus be able to form a salt bridge that mimics a disulfide bond. At low pH formation of a salt bridge might be prevented due to the protonation of asparate. Indeed, the induction profile supported by CadC_C208D,C272K was comparable to wild-type CadC (Figure 4). These data imply that in CadC_C208D,C272K the charged amino acids are able to form a salt bridge that takes over the function of the disulfide bond. In contrast, cells producing CadC_C208K,C272D exhibited a deregulated induction pattern (Figure 4). This result suggested that in this construct salt bridge formation was prevented and therefore the replacements of the cysteines against charged amino acids had the same effect as the disruption of the disulfide bond by alanine replacements.

Importantly, the wild-type like regulation pattern of CadC_C208D,C272K offered the unique opportunity to generate a functional cysteine-free CadC variant required as prerequisite for site-specific labeling studies in future. As expected, the regulation pattern of cells producing the cysteine-free derivative CadC_C172A,C208D,C272K was almost comparable to cells producing the wild-type protein (Figure 4). These data indicate that a salt bridge can take over the function of the disulfide bond in CadC.

### The disulfide bond in CadC affects the interaction between sensor and co-sensor

**Figure 5:**
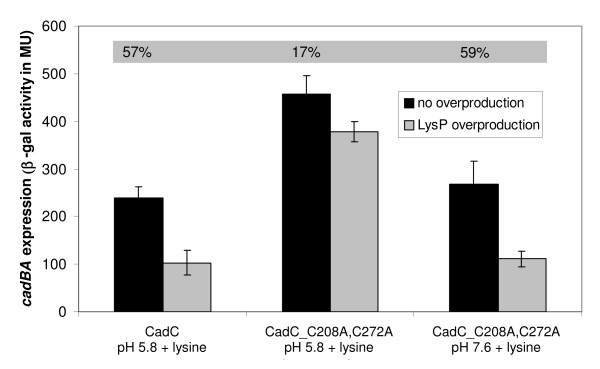
**Influence of LysP overproduction on CadC-mediated *cadBA *expression**. Reporter gene assays were performed with *E. coli *EP314 (*cadC*::Tn*10*; *cadA'*::*lacZ *fusion) which was co-transformed with plasmid-encoded *cadC *or *cadC_C208A,C272A *and with a second plasmid carrying the *lysP *gene (pBAD33-lysP). Cells were cultivated under microaerobic conditions in minimal medium at pH 5.8 or pH 7.6 in the presence of 10 mM lysine at 37°C to mid-logarithmic growth phase, and harvested by centrifugation. When indicated, overproduction of LysP was induced by addition of 0.2% (w/v) arabinose. The activity of the reporter enzyme β-galactosidase was determined [43] and served as a measurement for *cadBA *expression. Shaded numbers display the degree of inhibition of *cadBA *expression by LysP overproduction. It should be noted that wild-type CadC activates *cadBA *expression only at pH 5.8. Error bars indicate standard deviations of the mean for at least three independent experiments.

CadC activity is regulated by the two stimuli pH and lysine. CadC derivatives with a replacement of the periplasmic cysteines by alanine were inactive at pH 7.6 in the absence of lysine (Figure 1). Obviously, the inhibitory effect of LysP on the CadC derivatives was strong enough to prevent *cadBA *expression at pH 7.6. However, it remained unclear, why these CadC derivatives activated *cadBA *expression at low pH in the absence of lysine despite of the inhibitory effect of LysP on CadC. Thus the question arose, whether the disruption of the periplasmic disulfide bond alters the interaction between CadC and LysP. To answer this question, the interplay between CadC and LysP was disturbed, simply by overproduction of LysP [11,19]. It is known, that LysP overproduction lowers wild-type *cadBA *expression significantly (57% reduction) (Figure 5). In contrast, CadC_C208A,C272A-mediated *cadBA *expression was slightly affected by LysP overproduction at pH 5.8 (17%), but severely affected at pH 7.6 (59%) (Figure 5). These results imply that the interaction between LysP and CadC_C208A,C272A is weaker at pH 5.8 than at pH 7.6, and in general weaker in comparison to wild-type CadC. Moreover, the weakened interaction between LysP and CadC_C208A,C272A might also account for the general higher ß-galactosidase activities measured for all derivatives with Cys replacements at positions 208 and/or 272 (Figures 1 and 5).

In another experiment CadC_C208A,C272A was introduced into the Δ*lysP *reporter strain *E. coli *EP-CD4 (*cadC*::Tn*10*, *cadA'*::*lacZ*, Δ*lysP*). In a *lysP*^- ^background, wild-type CadC activates *cadBA *expression in a lysine-independent, but pH-dependent manner [11,19]. As expected, in the *lysP*^- ^background, CadC_C208A,C272A induced *cadBA *expression lysine- and pH-independently revealing that LysP is responsible for the inhibition of CadC_C208A,C272A in the absence of lysine at pH 7.6 (data not shown). As discussed below, these experiments revealed that CadC without a disulfide bond is transformed into a semi-active state with respect to both the pH and the lysine stimuli.

### Periplasmic disulfide oxidoreductases have no major influence on CadC activation

The results described above led to the hypothesis that at physiological pH CadC contains a disulfide bond which is reduced at low pH. Opening and formation of disulfide bonds requires either the corresponding environment (oxidizing or reducing) or enzymes that catalyze these processes. Therefore, we analyzed whether periplasmic proteins known to be involved in formation and opening of disulfide bonds during the protein folding process such as the Dsb proteins [20] have an influence on CadC activation. Six gene deletion mutants were constructed lacking the disulfide bond-modifying proteins DsbA, DsbB, DsbC, DsbD, DsbG and CcmG (also known as DsbE). CcmG does not belong to the Dsb system, but is a membrane-anchored protein with a periplasmic thiol:disulfide oxidoreductase domain involved in cytochrome c biogenesis [21]. DsbA is a disulfide oxidase responsible for the formation of disulfide bonds and is recycled by the membrane protein DsbB [20]. DsbC is an isomerase that opens wrongly formed disulfide bonds and introduces the correct ones and as such also exhibits a reductase activity. DsbG is a non-essential isomerase that is able to substitute for DsbC, and seems to protect single cysteines from oxidation that are needed in a reduced state to be catalytically active [22]. Both, DsbC and DsbG, are recycled by DsbD. While DsbB and DsbD are membrane proteins, DsbA, DsbC and DsbG are soluble proteins located in the periplasm.

**Figure 6:**
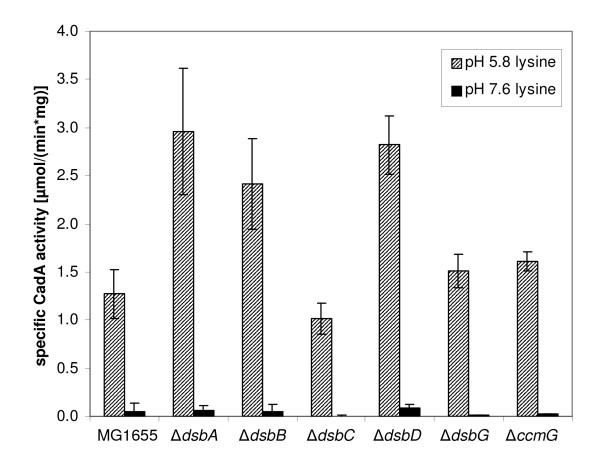
**Influence of periplasmic disulfide oxidoreductases on *cadBA *expression**. Single gene deletions of *dsbA*, *dsbB*, *dsbC*, *dsbD*, *dsbG *and *ccmG *were constructed in *E. coli *MG1655, and *cadBA *expression was monitored in the corresponding mutants by determination of CadA activity. Cells were cultivated under microaerobic conditions in minimal medium at pH 5.8 or pH 7.6 in the presence of 10 mM lysine. The specific CadA activity was determined [44] and is given in μmol/(min*mg protein). Error bars indicate standard deviations of the mean for at least three independent experiments.

Mutants of *E. coli *MG1655 each lacking a single *dsb *gene were grown at pH 5.8 and 7.6 in the presence of external lysine, and lysine decarboxylase (CadA) activity was determined as a measurement for the expression level of *cadBA *and thus of the functionality of CadC (Figure 6). All strains tested exhibited a pH-dependent regulation that was comparable to the wild-type strain, though the fold-induction differed slightly in some mutants. Under inducing conditions (pH 5.8, lysine) CadA activity was more than twice as high in the mutant MG1655Δ*dsbA*, lacking the disulfide oxidase DsbA, as in the wild-type strain MG1655 [specific CadA activity of 2.96 μmol/(min*mg protein) instead of 1.27]. The same holds true for MG1655Δ*dsbB*, lacking the recycling protein for DsbA, and MG1655Δ*dsbD*, lacking the recycling protein for DsbC [specific CadA activity of 2.41 and 2.82 μmol/(min*mg protein), respectively].

## Discussion

Cysteines are one of the most rarely used amino acids in proteins of all organisms studied so far [23]. Conserved cysteines usually play crucial roles in the structure and function of proteins because of their ability to form intra- and intermolecular disulfide bonds and to coordinate transition metal ions [24]. Disulfide bonds are often linked to protein stabilization, ligand binding, dimerization and activation. Examples of this are the eukaryotic cytokine receptor GHR [25] and the OxyR transcriptional factor of *E. coli *[26]. The ArcB sensor kinase is another example for the participation of disulfide bond formation during signaling [27,28]. Each ArcB monomer contains two cytosolic cysteines. Quinone-dependent inhibition of ArcB autophosphorylation involves the formation of one or two intermolecular disulfide bonds under aerobic conditions. The *V. cholerae *activator of virulence gene expression ToxR contains two periplasmic cysteines that are important for the formation of an intramolecular disulfide bond as well as for the formation of intermolecular disulfide bonds between two ToxR molecules and between ToxR and ToxS [12,13]. ToxR binds to the DNA only in a dimeric form [7], and dimerization of full-length ToxR was shown *in vivo *[29]. However, an influence of environmental conditions (pH, osmolarity) on ToxR oligomerization and hence disulfide bond formation *in vivo *was not detected [14].

CadC contains three cysteines. Here, we show that cysteines located at positions 208 and 272 are important for the function of CadC, whereas C172 is dispensable. Two stimuli are needed to activate wild-type CadC: low pH and lysine. Replacements of C208 and/or C272 resulted in CadC derivatives that partially induced *cadBA *expression at pH 7.6 indicating the transformation of CadC into a semi-active state. This discovery and structural data, according to which these residues are in close proximity to each other [15], prompted experiments to monitor the redox state of the thiol groups of the periplasmic cysteines in CadC *in vivo*. Differential thiol trapping performed with CadC_C172A producing cells that were grown in minimal medium at pH 7.6 revealed that a disulfide bond is present in CadC. In contrast, at pH 5.8 the periplasmic cysteines of CadC were found to be in the reduced state. These data indicate that CadC contains a functionally important disulfide bond. It is important to note, that CadC is hardly a redox sensor. The differences in the *cadBA *expression level found for anaerobic and aerobic growth conditions are dependent on H-NS [6]. Therefore, it is proposed that the disulfide bond in CadC provides structural support for the switch of the sensor between the inactive and active state. This assumption is supported by the location of Cys208 within a flexible loop in the N-terminal subdomain [15].

The question arose, how the disulfide bond might be formed and opened *in vivo*. Enzymes responsible for these processes might be the periplasmic disulfide oxidoreductases of the Dsb system. CadA activity as indication for *cadBA *expression was monitored in single *dsb *and *ccmG *deletion mutants. However, none of these deletions altered the CadC-mediated induction profile. In all deletion mutants induction of *cadBA *expression was prevented at pH 7.6, and CadA activity was significantly increased at low pH. These data imply that none of these proteins was essential for the formation or opening of the disulfide bond in CadC. It is worth mentioning, that we found an elevated CadA activity in the *dsbA *(encoding a disulfide oxidase)*, dsbB *(encoding a protein that regenerates DsbA) and *dsbD *(encoding a recycling enzyme for an isomerase/reductase) deletion mutants. DsbA/DsbB are responsible for the introduction of disulfide bonds in newly synthesized proteins, thus their lack might support a higher probability of CadC molecules without a disulfide bond and thus the increased CadA activity. The role of DsbD in CadC activation remains unknown. Nevertheless, either these enzymes are functionally redundant, or the spontaneous oxidation by oxygen or low molecular compounds might be responsible for the formation of a disulfide bond in CadC. *cadC *belongs to the genes/operons with the shortest half-lives of the mRNA [30]. Based on this result and our finding of a transient activation of CadC [31], we speculate that there is a rapid turnover of CadC and that the disulfide bond is preferentially introduced during *de novo *synthesis of CadC. The periplasm is accessible for oxygen and therefore allows the spontaneous oxidation of two neighboring cysteines in proteins [32,33]. Expression of the *cadBA *operon is induced at low pH, and the induction level is higher in the absence of oxygen [34]. Under these conditions the oxidation of cysteines to cystine is minimized due to the lack of oxygen as well as the surplus of protons which prevents the formation of thiolate anions, the prerequisite for disulfide bond formation [35]. Thus, this shift in the external conditions already dramatically reduces the probability to form a disulfide bond in CadC. Based on these results it is suggested that under non-inducing conditions (pH 7.6) a disulfide bond in the periplasmic domain holds the sensor in an inactive state. Under inducing conditions (pH 5.8) formation of the disulfide bond is either prevented or the disulfide bond is opened by a still unknown mechanism. Concomitantly, CadC undergoes conformational changes due to the protonation of negatively charged amino acids located in a patch at the CadC dimer interface [10].

This proposal is in accordance with the finding that the disulfide bond could be mimicked by a salt bridge. When C208 was replaced with an aspartate and C272 with a lysine, a CadC derivative was generated that supported *cadBA *expression comparable to the wild-type protein. Functional substitution of a disulfide bond by a salt bridge in CadC requires formation of the salt bridge at pH 7.6, which is conceivable (aspartate deprotonated, lysine protonated), and an opening of the salt bridge, which might depend on the protonation of aspartate at low pH [36,37]. In contrast, a CadC derivative in which the cysteines were replaced by the same charged amino acids but at opposite positions (CadC_C208K,C272D) caused deregulation of *cadBA *expression. It is suggested that a salt bridge was not formed in this derivative due to an unfavorable orientation of the amino acid side chains to each other.

**Figure 7:**
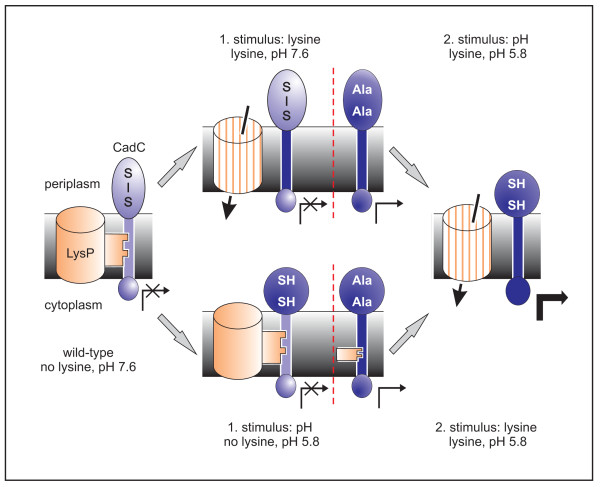
**Model of the lysine- and pH-dependent activation of wild-type CadC and CadC_C208A,C272A**. The different transcription activities are indicated by the arrows below CadC. Under non-inducing conditions (no lysine, pH 7.6) CadC-mediated *cadBA *expression is inhibited by two mechanisms, the interaction with LysP and a disulfide bond in the periplasmic domain. CadC with a disulfide bond remains inactive even when the interaction with LysP is released in the presence of lysine (lysine, pH 7.6). A shift to low pH causes conformational changes and prevents formation of a disulfide bond (lysine, pH 5.8). In the absence of lysine, CadC activity is blocked by the interplay with LysP (no lysine, pH 5.8). In CadC_C208A,C272A formation of a disulfide bond is prevented by amino acid replacements. As consequence, this derivative displays semi-active states at pH 7.6 in the presence of lysine (lysine, pH 7.6), and at pH 5.8 in the absence of lysine (no lysine, pH 5.8). See text for further details.

The results obtained in this study illuminate the activation mechanism, specifically the sequential events to transform CadC into an active form (Figure 7). Derivative CadC_C208A,C272A induced *cadBA *at pH 7.6, however, its activity further increased at pH 5.8. Thus, the lack of the disulfide bond seems to be only one part of the pH-dependent structural transitions in CadC. Whether reduction of the cysteines is a prerequisite for or a consequence of additional conformational changes cannot be decided yet. Nevertheless, CadC without a disulfide bond is held in a semi-active state. This derivative also induces *cadBA *expression at low pH regardless of the lysine concentration. This result suggests that the interaction between LysP and a CadC derivative without a disulfide bond is weaker in comparison to the wild-type. In agreement, CadC lacking the periplasmic cysteines is hardly subject to LysP-mediated inhibition in cells that overproduce LysP. Our experimental data also revealed that the interaction between LysP and CadC is stronger at pH 7.6.

Based on these results, a two step activation mechanism for CadC is proposed (Figure 7). Under non-inducing conditions (no lysine, pH 7.6) CadC-mediated *cadBA *expression is inhibited by two mechanisms. At pH 7.6 a disulfide bond is formed, and CadC is in an inactive form. Moreover, CadC is inhibited through the interplay with the lysine permease LysP in the absence of lysine [11]. CadC with a disulfide bond remains inactive even when the interaction with LysP is released in the presence of lysine (lysine, pH 7.6). Exposure of CadC to low pH is accompanied by conformational changes and reduction of the cysteines resulting in an active CadC (lysine, pH 5.8). Alternatively, at low pH in the absence of lysine, CadC is still locked in an inactive conformation due to the interplay with LysP (no lysine, pH 5.8). The presence of lysine suspends the interaction with LysP, and CadC is transformed into the active state (lysine, pH 5.8).

In CadC_C208A,C272A formation of a disulfide bond is prevented by amino acid replacements (Figure 7). As consequence, this derivative displays semi-active states at pH 7.6 in the presence of lysine (lysine, pH 7.6) or at low pH in the absence of lysine (no lysine, pH 5.8). Additional pH-dependent conformational changes or the presence of lysine are required to fully activate this CadC derivative (lysine, pH 5.8).

## Conclusion

Previously, it was proposed that the two stimuli, lysine and low pH, affect CadC activation independently from each other [38]. Here, we gained new insights into the molecular mechanism how CadC processes these stimuli, particularly that a disulfide bond is involved in the function of CadC.

## Methods

### Bacterial strains and growth conditions

Strains and plasmids are listed in Tables 1 and 2. *E. coli *JM109 served as carrier for the plasmids described. *E. coli *BL21(DE3)pLysS was used for expression of *cadC *and *cadC *derivatives from the T7 promoter. *E. coli *EP314 and EP-CD4 were complemented with plasmids (pET16b) encoding *cadC *and its derivatives, and used for *cadBA *transcriptional analysis. *E. coli *EP314 and EP-CD4 carry a *cadA'*::*lacZ *fusion and an inactivated *cadC*. Additionally, EP-CD4 is also *lysP*^-^. Overproduction of LysP was performed in *E. coli *EP314 transformed with plasmid pBAD33-lysP by inducing the arabinose promoter with 0.2% (w/v) arabinose. *E. coli *MG1655 was used for construction of gene deletion strains. *E. coli *strains were grown in Luria-Bertani (LB) medium [39] for strain maintenance and protein overproduction. To probe signal transduction *in vivo*, cells of *E. coli *EP314 transformed with the indicated plasmids were grown in minimal medium [40]; the phosphate buffer of the medium was adjusted to either pH 5.8 or pH 7.6. Lysine was added at a concentration of 10 mM. For selection of plasmid-containing cells appropriate antibiotics were added at concentrations of 100 μg/ml (ampicillin sodium salt), 50 μg/ml (kanamycin sulfate) and 34 μg/ml (chloramphenicol).

**Table 1:** Bacterial strains used in this study.

Strain	Relevant genotype	Source or reference
*E. coli *JM109	*recA1 endA1gyrA96 thi-1 hsdR17 *(*r*_K_^- ^*m*_K_^+^)	Stratagene
	*supE44 relA1Δ*(*lac-proAB*) [F' *traD36 proAB*	
	*lacI*^q^ZΔM15]	

*E. coli *BL21(DE3)pLysS	F^- ^*ompT r*^-^_B _*m*^-^_B _*dcm gal tonA *(DE3) pLysS (Cm^R^)	[46]

*E. coli *EP314	W3110 Δ*(lacIOPZYA) exa-*1::Mu	[19]
	dI*1734 *Km *lac) *in *cadA*] *cadC1*::Tn*10*	

*E. coli *EP-CD4	*E. coli *EP314 *lysP*::Cm	This work

*E. coli *MG1655	K12 reference strain	[47]

*E. coli *MG1655Δ*dsbA*	*E. coli *MG1655 Δ*dsbA*::Kan	This work

*E. coli *MG1655Δ*dsbB*	*E. coli *MG1655 Δ*dsbB*::Kan	This work

*E. coli *MG1655Δ*dsbC*	*E. coli *MG1655 Δ*dsbC*::Cm	This work

*E. coli *MG1655Δ*dsbD*	*E. coli *MG1655 Δ*dsbD*::Kan	This work

*E. coli *MG1655Δ*dsbG*	*E. coli *MG1655 Δ*dsbG*::Kan	This work

*E. coli *MG1655Δ*ccmG*	*E. coli *MG1655 Δ*ccmG*::Kan	This work

**Table 2:** Plasmids used in this study.

Plasmid	Relevant genotype	Source or reference
pET16b	Expression vector, Ap^r^	Novagen

pET16b-cadC	*cadC *in pET16b	[6]

pET16b-cadC_C172A	Amino acid exchange C172A in *cadC*,	This work
	*cadC_C172A *in pET16b	

pET16b-cadC_C208A	*cadC_C208A *in pET16b	This work

pET16b-cadC_C272A	*cadC_C272A *in pET16b	This work

pET16b-cadC_C172A,C208A	*cadC_C172A,C208A *in pET16b	This work

pET16b-cadC_C172A,C272A	*cadC_C172A,C272A *in pET16b	This work

pET16b-cadC_C208A,C272A	*cadC_C208A,C272A *in pET16b	This work

pET16b-cadC_C172A,C208A,C272A	*cadC_C172A,C208A,C272A *in pET16b	This work

pET16b-cadC_C208D,C272K	*cadC_C208D,C272K *in pET16b	This work

pET16b-cadC_C208K,C272D	*cadC_C208K,C272D *in pET16b	This work

pET16b-cadC_C172A,C208D,C272K	*cadC_C172A,C208D,C272K *in pET16b	This work

pBAD33	Expression vector, Cm^r^	[48]

pBAD33-lysP	*lysP *in pBAD33	[11]

Site-directed mutants are designated as follows: The one letter code is used, followed by a number indicating the position of the amino acid in wild-type CadC.

The sequence is followed by a second letter denoting the amino acid replacement at this position.

### Generation of plasmids and strains

All *cadC *derivatives were constructed by polymerase chain reaction (PCR) with mismatch primers either by single step or by two step PCR [41]. To facilitate construction, a *cadC *gene with two additional unique restriction sites was employed [11]. All site-specific mutations were directed by synthetic oligonucleotide primers containing the required nucleotide exchanges. PCR fragments were cloned into the expression vector pET16b with the restriction enzymes *Nde*I and *Bam*HI so that all constructs carried the sequence encoding an N-terminal His-Tag of 10 histidine residues. *E. coli *EP-CD4, *E. coli *MG1655Δ*dsbA*, *E. coli *MG1655Δ*dsbB*, *E. coli *MG1655Δ*dsbC*, *E. coli *MG1655Δ*dsbD*, MG1655Δ*dsbG *and MG1655Δ*ccmG *were constructed by deleting the genes *lysP, dsbA, dsbB, dsbC, dsbD*, *dsbG *and *ccmG*, respectively, via the Quick & Easy *E. coli *Gene Deletion Kit (Gene Bridges) according to the manufacturer's instructions.

### Differential thiol trapping of CadC *in vivo*

The thiol/disulfide state of the periplasmic cysteines of CadC was monitored *in vivo *by differential thiol trapping according to [16]. The procedure was modified as follows: *E. coli *BL21(DE3)pLysS carrying one of the plasmids pET-CadC-C172A, pET-CadC-C172A,C208A or pET-CadC-C172A,C208A,C272A was grown in phosphate buffered minimal medium with a pH of 7.6 or 5.8 to an OD_600 _of 0.5. Subsequently, overproduction of the CadC derivatives was induced by addition of 0.5 mM IPTG. After an additional hour of growth at 37°C, the OD_600 _was adjusted to 1, and 5 mM iodoacetamide (dissolved in 0.1 M Tris) was added to 1 ml cell suspension. At pH 7.6, incubation was performed for 15 min (37°C), at pH 5.8 the incubation time was prolonged to 150 min to compensate the lower alkylation rate of iodoacetamide at low pH. This first alkylation procedure irreversibly modified all free thiol groups directly in the living cells. Subsequently, cells were harvested into 100 μl ice-cold 100% (w/v) trichloric acid (TCA) and stored on ice for at least 30 min. The TCA treated cells were centrifuged (16.000 g, 4°C, 15 min), and the resulting pellet was washed with 200 μl of ice-cold 10% (w/v) TCA followed by a wash with 100 μl of ice-cold 5% (w/v) TCA. The supernatant was removed completely, and the pellet was resuspended in 100 μl of denaturing buffer [6 M urea, 200 mM Tris-HCl (pH 8.5), 10 mM EDTA, 0.5% (w/v) SDS] supplemented with 10 mM DTT to reduce disulfide bonds. After one hour of incubation in the dark (37°C, gentle agitation at 1300 rpm), 10 μl ice-cold 100% (w/v) TCA was added, and the sample was stored on ice for at least 30 min. After centrifugation, the resulting pellet was again washed with 200 μl of ice-cold 10% (w/v) TCA followed by a wash with 100 μl of ice-cold 5% (w/v) TCA. Finally, the pellet was resuspended in 50 μl of denaturing buffer containing 10 mM PEG-maleimide (Iris Biotech GmbH, Marktredwitz/Germany) to alkylate all newly reduced cysteines. The reaction (37°C, gentle agitation at 1300 rpm, in the dark) was stopped after one hour by addition of 5 μl ice-cold 100% (w/v) TCA. After precipitation on ice (30 min) and centrifugation, the pellet was washed first with 100 μl of 10% and then with 50 μl of 5% ice-cold (w/v) TCA. After removing the TCA, the pellet was washed twice with 500 μl acetone and resuspended in 50 μl denaturing buffer. Samples were mixed with non-reducing SDS-sample buffer and loaded onto 12.5% SDS-polyacrylamide gels [42]. CadC was detected by Western blot analysis [11].

### Analysis of intermolecular disulfide bonds

For the detection of intermolecular disulfide bonds, wild-type CadC and all available CadC derivatives with Cys replacements (CadC_C172A; CadC_C208A; CadC_C272A; CadC_C172A,C208A; CadC_C172A,C272A; CadC_C208A,C272A; CadC_C172A,C208A,C272A) were overproduced in *E. coli *BL21(DE3)pLysS, and membrane vesicles were prepared [11]. CadC-containing membrane vesicles [1 mg protein/ml in TG-buffer, 50 mM Tris/HCl, pH 7.5; 10% (v/v) glycerol] were treated with 0.2 mM copper phenanthroline at 25°C for 30 min. The reaction was stopped by addition of 10 mM EDTA. Samples were mixed with non-reducing SDS-sample buffer and loaded onto 7.5% (w/v) SDS-polyacrylamide gels [39]. CadC was detected by Western blot analysis [11].

### Measurement of CadC signal transduction activity *in vivo*

Signal transduction activity of different CadC derivatives *in vivo *was probed with a β-galactosidase based reporter gene assay as previously described [11]. Using a pET-based vector in combination with the reporter strain *E. coli *EP314 that does not possess a T7 polymerase resulted in a low expression that was sufficient to allow complementation but did not lead to overproduction of CadC which would result in stimulus-independent *cadBA *expression. β-galactosidase activity was determined from at least three independent cultures, and is given in Miller units (MU) calculated as described [43]. The activity of the lysine decarboxylase CadA as a measurement for *cadBA *expression was determined according to [44] with the following changes: for the assay cells corresponding to an optical density of 1 (600 nm) were resuspended in 20 mM potassium phosphate buffer (pH 5.6) and lysed by the addition of chloroform. One unit is defined as 1 μmol decarboxylated lysine produced per minute and specific activities were calculated for 1 mg of protein [μmol/(min*mg)]. Insertion of the CadC derivatives into the cytoplasmic membrane was analyzed after overproduction of CadC, isolation of membrane vesicles and subsequent Western blot analysis as previously described [11,45].

## Authors' contributions

LT, CK and KJ designed research experiments; AD performed experiments; LT performed experiments and analyzed data. LT and KJ wrote the manuscript. All authors have read and approved the final manuscript.
